# Multi-Objective Optimization of the Basic and Regenerative ORC Integrated with Working Fluid Selection

**DOI:** 10.3390/e24070902

**Published:** 2022-06-29

**Authors:** Yuhao Zhou, Jiongming Ruan, Guotong Hong, Zheng Miao

**Affiliations:** 1 Key Laboratory of Technology on Space Energy Conversion, Technical Institute of Physics and Chemistry, Chinese Academy of Sciences, Beijing 100190, China; yuhao-zhou@chder.com; 2 University of Chinese Academy of Sciences, Beijing 100049, China; 3 Huadian Electric Power Research Institute Co., Ltd., Hangzhou 310030, China; jiongming-ruan@chder.com; 4 Beijing Key Laboratory of Multiphase Flow and Heat Transfer for Low-Grade Energy Utilization, North China Electric Power University, Beijing 102206, China

**Keywords:** multi-objective optimization, working fluid selection, NSGA-II, regenerative ORC system, exergy efficiency, thermal efficiency, levelized energy cost

## Abstract

A multi-objective optimization based on the non-dominated sorting genetic algorithm (NSGA-II) is carried out in the present work for the basic organic Rankine cycle (BORC) and regenerative ORC (RORC) systems. The selection of working fluids is integrated into multi-objective optimization by parameterizing the pure working fluids into a two-dimensional array. Two sets of decision indicators, exergy efficiency vs. thermal efficiency and exergy efficiency vs. levelized energy cost (LEC), are adopted and examined. Five decision variables including the turbine inlet temperature, vapor superheat degree, the evaporator and condenser pinch temperature differences, and the mass fraction of the mixture are optimized. It is found that the turbine inlet temperature is the most effective factor for both the BORC and RORC systems. Compared to the reverse variation of exergy efficiency and thermal efficiency, only a weak conflict exists between the exergy efficiency and LEC which tends to make the binary objective optimization be a single objective optimization. The RORC provides higher thermal efficiency than BORC at the same exergy efficiency while the LEC of RORC also becomes higher because the bare module cost of buying one more heat exchange is higher than the cost reduction due to the reduced heat transfer area. Under the heat source temperature of 423.15 K, the final obtained exergy and thermal efficiencies are 45.6% and 16.6% for BORC, and 38.6% and 20.7% for RORC, respectively.

## 1. Introduction

The low-medium temperature thermal energy widely exists in the conventional industry processes as waste heat and renewable energy areas, such as the solar thermal energy, geothermal heat, and biomass. The organic Rankine cycle (ORC) is a promising technology for converting low-medium heat into useful mechanical work [1,2,3]. Due to the advantage of relatively high efficiency, simple configuration, and ease of maintenance, the ORC system has attracted considerable attention in the past two decades. Plenty of research has been reported concerning the issues including the screening of working fluids [4], optimization of cycle parameters [5], development of advanced cycle configurations [6,7], testing of the prototypes [8,9], simulation of the designed unit [10,11]. 

The screening of working fluid is always a key concern for the study and design of the ORC system as the properties of the working fluid impose a strong effect on the cycle performance. Numerous works have been reported on this aspect, most of which focus on the comparison of ORC thermodynamic performance with several working fluids chosen randomly or by their experience, then suggest one or more working fluids among the candidates. Some researchers tried to develop the selection criteria of working fluid for the ORC system to preliminary screen the candidates from numerous substances just according to the thermophysical properties of the working fluid rather than the complicated thermodynamic calculation. Györke et al. [12] proposed a novel classification method of pure working fluids for ORC based on the existence and relative location of some characteristic points of the vapor-liquid coexistence curve in *T-s* diagram to find the thermodynamically optimal working fluid for a given heat source. In addition, researchers have noticed that critical temperature could be a clear indicator to primarily screen working fluids. For the pure working fluid, some scholars [13,14] found that the optimal critical temperature should be 30–50 K lower than the heat source inlet temperature. Vetter et al. [15] claimed that the optimal critical temperature should be 0.8 times of the heat source inlet temperature. Zhai et al. [16] reported a linear relationship between the critical temperature and the heat source inlet temperature. For the mixtures, both the thermal match in the evaporator and the condenser should be considered. Zhao et al. [17] found that the heat source inlet temperature has a prominent influence on the composition of zeotropic mixtures. In our former works [18,19], we have proposed the thermodynamic criteria to screen mixture working fluid for the ORC system driven by the open and closed heat source. Correlations related to the optimal critical temperature and condensation temperature glide were proposed. The case study proved that the optimal thermodynamic and thermo-economic performance can be simultaneously obtained for the ORC system with the proposed selection criteria. Up to now, most works related to the selection of working fluids are single-objective oriented. The highest thermal efficiency, exergy efficiency or thermo-economic performance is expected. However, in most cases, the trade-off between several performance indicators should be considered according to the usage scenarios and the decision maker’s preference. It is a typical multi-objective optimization issue. 

Typically, system parameters optimization is the necessary approach for improving the system performance. Various criteria could be set as the objectives, including the thermodynamic indicators (the net power output, thermal efficiency, exergy efficiency), the economic indicators (heat transfer area, total investment, payback period), and the thermo-economic indicators (levelized energy cost (LEC), heat transfer area per unit power (APR), levelized cost of electricity (LCOE)) [20,21,22]. Conflicts may exist between different objectives. For example, the increase in the evaporation temperature leads to the increase in the thermal efficiency yet the decrease in the net power output. In this condition, the multi-objective optimization algorithm is needed to achieve the Pareto frontier solutions, which is a set of trade-off solutions considering these objectives. All of the solutions on the Pareto frontier are optimal. The final preferred solution is then determined by the concept of ‘weight’, which reflects the preference of the decision-maker. Yang et al. [23] carried out the binary-objective optimization of low-GWP alternatives to R245fa for the ORC system. The results showed that both the cycle thermal efficiency and the LECT (total savings of levelized electricity cost) are sensitive to evaporator outlet temperature. Comparing the maximum LECTs, the R1224yd(Z), R1234ze(Z), and R1233zd(E) can improve the LECT by 16%, 9.2%, and 13.5% higher than R245fa, respectively. Hu et al. [24] also compared the ORC performance using hydrofluorolefins (HFOs) through multi-objective optimization and reported that the evaporation temperature is the most relevant decision variable and R1234ze(E) is optimal to offer the largest power output with the weight of economic performance (W_1_ < 0.2). Fergani et al. [25] performed an exergy-based multi-objective optimization of an ORC with zeotropic mixtures and found that the mixtures could provide a significant improvement in energetic, economic, and environmental performances. Xia et al. [26] proposed a method combing multi-objective optimization with improved grey relational analysis (GRA) to select working fluids for the dual-loop ORC system. They claimed that cyclohexane/butane has the best comprehensive performance among 27 alternatives and the boiling temperature is a criterion of fluid selection for the dual-loop ORC system. Hundreds of works about multi-objective optimization have been reported to select the working fluid, optimize system parameters, or compare configurations, among which most work repeat the calculation for every working fluid. It will lead to a rapid increase in the computational load, especially when the mixture is used. There is a scientific gap to integrate the selection of working fluid with the multi-objective optimization to achieve the optimal system parameters and corresponding working fluid simultaneously. 

Exploring more effective configurations of the ORC system is another way to improve the system performance [27]. The regenerative ORC (RORC) is the most attractive configuration developed by installing an internal heat exchanger (IHE) into the BORC to recover surplus heat of the turbine exhaust and preheat the liquid working fluid at the pump outlet. The study of Groniewsky and Wagner [28] proved that the interaction existed between the working fluid selection and the cycle configuration development. The RORC is not superior over the BORC. Therefore, both the simple and the regenerative topologies should be investigated in the earliest stages of the design process. Nondy and Gogoi [29] presented the multi-objective optimization to compare different ORC configurations for waste heat recovery by the Pareto Envelope-based Selection Algorithm-II (PESA-II). The Regenerative Recuperative ORC was suggested as it shows 16.19% and 15.33% higher net power and exergy efficiency compared to the BORC, while the system cost rate is 1.68% low. Feng et al. [30] conducted thermo-economic multi-objective optimization by using the non-dominated sorting genetic algorithm to compare RORC and BORC. They found that the optimum exergy efficiency and LEC for the Pareto-optimal solution of the RORC are 8.1% and 21.1% higher than that of the BORC. Hou et al. [31] performed the multi-objective optimization for a novel combined supercritical CO_2_ recompression cycle and RORC using zeotropic mixture. Several zeotropic mixtures are parameterized and used as a decision variable to participate in the multi-objective optimization process to obtain the optimal zeotropic mixture. This approach could be a reference to carry out the working fluid selection and parameter optimization together.

Based on the literature review, in the present study, the multi-objective optimization based on the NSGA-II is carried out for the BORC and RORC systems. The selection of working fluid is integrated into multi-objective optimization by parameterizing the names of pure working fluids into the two-dimensional array of numbers which could be treated as two decision variables in NSGA-II and optimized together with other five variables including the turbine inlet temperature, vapor superheat degree, the evaporator and condenser pinch temperature differences, and the mass fraction of the mixture. Two pairs of objectives, exergy efficiency vs. thermal efficiency and exergy efficiency vs. LEC are adopted and examined.

## 2. System Description

**Figure 1:**
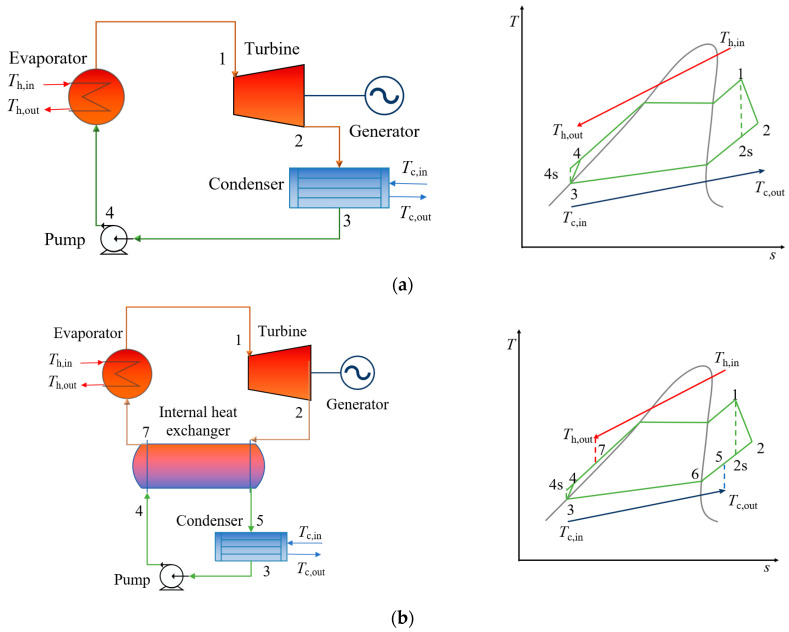
Schematic and *T-s* diagram of (**a**) the BORC system, and (**b**) the RORC system.

The *T-s* and schematic diagrams of the BORC and RORC are shown in Figure 1. The working fluid is heated in the evaporator and then expands in the expander to generate mechanical work. The exhaust vapor is condensed into a liquid phase in the condenser and then pressurized by the pump to run the cycle continually. For the RORC system, the exhaust vapor of the turbine first enters the regenerator to preheat the working fluid from the pump. The heat source fluid flow through the evaporator is air with a mass flow rate of 30 kg/s and the inlet temperature from 150 °C to 250 °C with the step of 50 °C. The cooling fluid in the condenser is water with an inlet temperature of 20 °C.

The general assumptions adopted in the present work are as follows:

1. The proposed system operates at a steady state;
2. The maximum evaporation pressure is restricted below 90% of the working fluid critical pressure to ensure the safe operation of the ORC system;
3. The ambient pressure and temperature are 101.3 kPa and 293.15 K, which is the reference state for the exergy analysis;
4. The isentropic efficiencies of the expander and pump are assumed as 0.85;
5. The heat exchangers are treated as externally adiabatic devices.

## 3. Mathematical Model

In the self-developed simulation program, the REFPROP 9.1 database was used to derive the thermodynamic properties of the working fluids. A pinch point temperature difference (PPTD) was specified for the heat transfer process to realize the establishment of the cycle configuration and the calculation of cycle performance.

### 3.1. Thermodynamic Analysis

The thermodynamic analysis was carried out according to the first and second laws of thermodynamics. The heat flux between the heat source and the working fluid in the evaporator of both the BORC and RORC can be given as:

(1) $$Q_{h}={\dot{m}}_{h}\left(h_{h,\mathrm{in}}-h_{h,\mathrm{out}}\right)={\dot{m}}_{\mathrm{wf}}\left(h_{1}-h_{4}\right)$$

where ${\dot{m}}_{h}$ and ${\dot{m}}_{\mathrm{wf}}$ are the mass flow rate of the heat source fluid and working fluid, $h_{h,\mathrm{in}}$, $h_{h,\mathrm{out}}$, $h_{1}$, and $h_{4}$ are the specific enthalpies of the heat source and working fluid at the inlet and outlet of the evaporator. The subscript of the formula corresponds to the labels in Figure 1. 

For the RORC, the heat flux in the IHE can be expressed as:

(2) $$Q_{h}={\dot{m}}_{\mathrm{wf}}\left(h_{2}-h_{5}\right)={\dot{m}}_{\mathrm{wf}}\left(h_{7}-h_{4}\right)$$

The effectiveness of the IHE is defined as:

(3) $$\eta_{\mathrm{IHE}}=\frac{\left(T_{2}-T_{5}\right)}{\left(T_{2}-T_{4}\right)}$$

And $\eta_{\mathrm{IHE}}$ is set as 0.8 in the present study.

The net output power of the ORC system is the difference between the power of the turbine and the power consumed by the pump:

(4) $$W_{\mathrm{net}\;}=W_{\mathrm{tur}\;}-W_{\mathrm{pump}\;}$$

The power consumed by the pump $W_{\mathrm{pump}\;}$ in the BORC and RORC can be derived as follows:

(5) $$W_{\mathrm{pump}\;}={\dot{m}}_{\mathrm{wf}}\left(h_{4}-h_{3}\right)={\dot{m}}_{\mathrm{wf}}\left(h_{4s}-h_{3}\right)/\eta_{\mathrm{pump}\;}$$

The power of the turbine in both the BORC and RORC can be expressed as:

(6) $$W_{\mathrm{tur}}={\dot{m}}_{\mathrm{wf}}\left(h_{1}-h_{2}\right)={\dot{m}}_{f}\left(h_{1}-h_{2s}\right)\eta_{\mathrm{tur}\;}$$

where $\eta_{\mathrm{pump}\;}$ and $\eta_{\mathrm{tur}\;}$ are the isentropic efficiency of the pump and turbine.

The thermal efficiency of the system can be calculated by

(7) $$\eta_{I}=\frac{W_{\mathrm{net}\;}}{Q_{h\;}}$$

The exergy efficiency of the cycle is expressed as:

(8) $$\eta_{\mathrm{II}}=\frac{W_{\mathrm{net}\;}}{E_{\mathrm{in}\;}}$$

where $E_{\mathrm{in}\;}$is the total exergy entering the system:

(9) $$E_{\mathrm{in}}={\dot{m}}_{h}\left(h_{h,\mathrm{in}}-h_{0}-T_{0}\left(s_{h,\mathrm{in}}-s_{0}\right)\right)$$

It is noted that several definitions of exergy efficiency exist in the literature, and the exergy efficiency defined in the present work in Equations (6) and (7) considers only the total exergy entering the system but not the difference between the exergy entering and leaving the system. For a specified heat source at a certain temperature, the $E_{\mathrm{in}\;}$ is fixed, thus the exergy efficiency of the cycle is in proportion to the net power of the cycle. In such a way, these two variables cannot be selected as the individual target of the multi-objective optimization algorism. 

### 3.2. Thermo-Economic Analysis

The incorporation of thermodynamic and economic analysis provides the development of a cost-effective ORC system. The modular costing method, which is commonly used in the chemical industry, is adopted in the present work to evaluate the cost of each piece of equipment in the ORC system [32,33,34,35]. The bare module equipment cost are calculated as follows [36]:

(10) $$C_{\mathrm{bm}}=C_{p}^{0}F_{\mathrm{bm}}$$

where $C_{p}^{0}$ is the procurement cost of the equipment at ambient pressure and made of common materials, and $F_{\mathrm{bm}}$ is the bare module factor considering the influence of the pressure and materials on the cost. These two parts are given as [18,35]:

(11) $$\log_{10}C_{p}^{0}=K_{1}+K_{2}\log_{10}\left(Y\right)+K_{3}{\left[\log_{10}\left(Y\right)\right]}^{2}$$

(12) $$F_{\mathrm{bm}}=B_{1}+B_{2}F_{m}F_{p}$$

(13) $$\log_{10}F_{p}=C_{1}+C_{2}\log_{10}\left(P\right)+C_{3}{\left[\log_{10}\left(P\right)\right]}^{2}$$

where *Y* represents the component capacity and could be the heat transfer area of heat exchangers and the power of the turbine/pump. $F_{m}$ and $F_{p}$ are the material factor and pressure factor, respectively. $B_{1}$ and $B_{2}\;$ are the coefficients related to the types of components. The coefficients $K_{1}$–$K_{3}$ $B_{1}$–$B_{2}$, $F_{m}$, $C_{1}$–$C_{3}$, are given in Table 1 [18,34,35,37].

Based on the bare module cost of each component, the total cost of the ORC system is obtained:

(14) $$C_{\mathrm{tot}}=\left(C_{\mathrm{bm},\mathrm{eva}}+C_{\mathrm{bm},\mathrm{reg}}+C_{\mathrm{bm},\exp}+C_{\mathrm{bm},\mathrm{con}}+C_{\mathrm{bm},pump}\right)\frac{CEPCI_{2020}}{CEPCI_{2001}}$$

where $C_{\mathrm{bm},\mathrm{reg}}$ is none of the BORC. CEPCI (Chemical Engineering Plant Cost Index) is calculated according to the data in 2001 and 2020 [38,39], $CEPCI_{2001}$ = 397 and $CEPCI_{2020}$ = 668. It is noted that the cost of working fluid is neglected in this study as many works have revealed that it attributes to less than 1% of the total cost [40,41].

Up to this point, the LEC is chosen as the thermo-economic indicator, which considers both the thermodynamic and economic performance of the ORC system:

(15) $$LEC=\left(CRF\cdot C_{\mathrm{tot}}+C_{\mathrm{om}}\right)/\left(t_{\mathrm{op}}\cdot W_{\mathrm{net}}\right)$$

(16) $$C_{\mathrm{om}}=1.5\%C_{\mathrm{tot}}$$

where $C_{\mathrm{om}}$ is the maintenance cost of the system, and the annual operation hour $t_{\mathrm{op}}$ is set as 8000 h. The CRF is the capital recovery factor, given as:

(17) $$CRF=i{\left(1+i\right)}^{\mathrm{LT}}/\left[{\left(1+i\right)}^{\mathrm{LT}}-1\right]$$

where the equipment lifetime LT and the interest rate i are set as 20 years, 5% [31,42].

### 3.3. Heat Exchanger Model

The heat transfer area needs to be calculated to estimate the cost of heat exchangers according to Equation (9). The shell-tube heat exchangers with the counter-flow arrangement are used in the present work. The heat transfer area is calculated using the classical logarithmic mean temperature difference method (LMTD):

(18) $$T_{p}=\frac{T_{p,\max}-T_{p,\min}}{\ln\frac{T_{p,\max}}{T_{p,\min}}}$$

(19) $$A=\frac{Q}{KT_{p}}$$

where K is the overall heat transfer coefficient and is given as:

(20) $$K=\frac{1}{\frac{1}{\alpha_{\mathrm{in}}}\frac{d_{0}}{d_{\mathrm{in}}}+\frac{d_{0}}{2\lambda }\ln\frac{d_{0}}{d_{\mathrm{in}}}+\frac{1}{\alpha_{0}}}$$

(21) $$\alpha =\frac{\lambda Nu}{d}$$

The $\alpha_{\mathrm{in}\;}$ and $\alpha_{0}$ are the convective heat transfer coefficients at the inside and outside surface of the tube, the $d_{0}$ and $d_{\mathrm{in}\;}$ indicates the outer and inner diameter of the heat exchanger tube. The heat source fluid at the shell side of the evaporator is air and the cooling fluid at the shell side of the condenser is water. Heat transfer fluids in the regenerator are the liquid and vapor of the working fluid. For the single-phase convective heat transfer at the shell side, the Kern correlation is used [43]:

(22) $$Nu=0.36Re^{0.55}{\mathrm{Pr}}^{0.33}$$

The Gnielinski correlation [44] is used for the single phase convective heat transfer in the tube side:

(23) $$Nu=\frac{\left(f/8\right)\left(Re-1000\right)Pr}{1+12.7{\left(f/8\right)}^{0.5}\left({\mathrm{Pr}}^{2/3}-1\right)}$$

(24) $$f={\left[0.790ln\left(Re\right)-1.64\right]}^{-2}$$

Equations (22)–(24) are enough for the heat transfer calculation of the regenerator. The evaporator and condenser should also consider the boiling and condensation heat transfer. For the flow boiling in the two-phase region of the evaporator, the Gungor-Winterton correlation [45] is adopted to calculate the heat transfer coefficient $\alpha_{\mathrm{TP}}$ with the correction factors $F_{c}$ for mixture working fluids [46].

(25) $$\alpha_{\mathrm{TP}}=E\alpha_{L}$$

(26) $$E=1+3000{\left(BoFc\right)}^{0.86}+1.12{\left(\frac{x}{1-x}\right)}^{0.75}{\left(\frac{\rho_{L}}{\rho_{V}}\right)}^{0.41}$$

(27) $$\alpha_{L}=0.023Re_{L}^{0.8}Pr_{L}^{0.4}\frac{k_{L}}{d_{i}}$$

(28) $$F_{c}={\left\{1+\left(\frac{\alpha_{\mathrm{id}}}{q}\right)\Delta T_{\mathrm{evp}}\left[1-exp\left(\frac{-qB_{0}}{\rho_{L}\Delta H_{\mathrm{vap}}\beta_{L}}\right)\right]\right\}}^{-1}$$

where Bo is the boiling number, $\alpha_{\mathrm{id}}$ has the same correlation of $\alpha_{L}$ but using the mixture’s thermophysical properties to calculate the $Re_{L}$, $Pr_{L}$, and $k_{L}$. $\Delta T_{\mathrm{evp}}$ is the temperature glide during boiling process; $B_{0}$ is the ratio factor, and $\beta_{L}$ is the mass transfer coefficient. When the pure working fluid is used, the $F_{c}$ becomes unity.

For the condensation process in the condenser, the Shah correlation [47] is used to derive the heat transfer coefficient with the correction approach proposed by Bell and Ghaly [48]:

(29) $$\alpha_{\mathrm{TP}}=\{\begin{array}{c} \alpha_{I},\;J_{V}\geq 0.98{\left(Z+0.263\right)}^{-0.62}\\ \alpha_{I}+\alpha_{\mathrm{Nu}},J_{V}\geq 0.98{\left(Z+0.263\right)}^{-0.62} \end{array}$$

(30) $$\alpha_{I}=\alpha_{L}\left(1+\frac{3.8}{Z^{0.95}}\right){\left(\frac{\mu_{L}}{14\mu_{V}}\right)}^{\left(0.0058+0.557p_{r}\right)}$$

(31) αNu=1.32ReL(−1/3)[ρL(ρL−ρV)gkL3μL2]1/3

(32) $$\frac{1}{\alpha_{\mathrm{mix}}}=\frac{1}{\alpha_{\mathrm{mono}\;}}+\frac{Y_{V}}{\alpha_{V}}$$

(33) $$Y_{V}=xC_{\mathrm{pv}}\frac{\Delta T_{\mathrm{con}}}{\Delta H_{\mathrm{vap}}}$$

where $\alpha_{\mathrm{mono}\;}$ is $\alpha_{\mathrm{TP}}$ but with the mixture’s thermophysical properties, and $\Delta T_{\mathrm{con}}$ is the condensation temperature glide.

### 3.4. Working Fluid Selection and Multi-Objective Optimization with NSGA-II

#### 3.4.1. Multi-Objective Optimization with NSGA-II

The ORC system, similarly to other practical engineering systems, has several indexes in its performance evaluation criteria, such as the output work, thermal efficiency, exergy efficiency, LEC, and other thermo-economic indexes. In most circumstances, many objectives conflict with one another. When two or more indexes are chosen as the objectives to optimize the system design or operation parameters, the improvement of one objective’s performance will lead to the degradation of another objectives’ performance. In this condition, the multi-objective optimization method is required to achieve the suboptimum solutions, which is a trade-off between the performance of each objective and given as the Pareto frontier solutions. 

The non-dominated sorting genetic algorithm (NSGA-II) is adopted in the present work, which is a classic multi-objective optimization method frequently used in complicated multi-objective optimization problems. The optimization objectives of NSGA-II can be expressed as [20,26]:

(34) $$miny=f\left(x\right)=\left[f_{1}\left(x\right),f_{2}\left(x\right),\ldots ,f_{j}\left(x\right)\right],j=1,2,\ldots ,M$$

(35) $$x=\left[x_{1},x_{2},\ldots ,x_{i}\right]$$

(36) $$\begin{array}{r} g_{k}\left(x\right)\leq 0,k=1,2,\ldots ,p\\ h_{z}\left(x\right)=0,z=1,2,\ldots ,q\\ x_{imin}\leq x_{i}\leq x_{i,max},i=1,2,\ldots ,n \end{array}$$

where *n* and *M* are the numbers of decision variables and objectives, *p* and *q* are the inequality and equality constraints.

#### 3.4.2. Decision-Making with TOPSIS

In order to apply the multi-objective optimization method to actual issues, one of the Pareto frontier solutions must be selected according to the preference. The TOPSIS method is widely used as the decision-maker. This approach normalizes the solutions and transforms them into a matrix. For each target, the ideal and non-ideal solutions are determined as the best case and the worst case. Then, the distance between each evaluation objective and the ideal and non-ideal solutions is calculated as follows [29],

(37) $$d_{i+}=\sqrt{\overset{N}{\underset{j=1}{\sum }}{\left(f_{ij}-f_{j}^{ideal}\right)}^{2}},\;\mathrm{for}\;i=1,2,\ldots ,m$$

(38) $$d_{i-}=\sqrt{\overset{N}{\underset{j=1}{\sum }}{\left(f_{ij}-f_{j}^{non\text{-}ideal}\right)}^{2}},\;\mathrm{for}\;i=1,2,\ldots ,m$$

The relative distance $C_{i}^{*}$ between each evaluation objective and the optimal solution is given as:

(39) $$C_{i}^{*}=\frac{d_{i-}}{d_{i-}+d_{i+}},\;\mathrm{for}\;i=1,2,\ldots ,m$$

The optimal solution should have the shortest distance from the ideal solution.

#### 3.4.3. Working Fluid Selection Integrated with NSGA-II

The selection of working fluid is crucial to the ORC system. The zeotropic mixture can optimize the heat transfer process and reduce exergy losses. For a typical multi-objective optimization of the ORC system, the working fluid is fixed, pure substance or their mixtures. If the working fluid needs to be selected, the multi-objective optimization algorithm should be repeated for every working fluid candidate. This would be a challenge for the mixtures as the computational load increases significantly as both the pure working fluid types and their concentration should be considered. In addition, it becomes more complicated for the decision-making algorithm to determine the optimal solution. In the present work, we want to integrate the selection of working fluid into the NSGA-II method to deal with the above issue. The proposed method is to treat the ASHRAE (American Society of Heating, Refrigerating, and Air-Conditioning Engineers) names of the pure substance into a variable to be optimized by numbering them. When the mixtures are used, three variables are set corresponding to two series of pure working fluids and their concentration. As soon as the random number is generated in the NSGA-II algorithm, it is rounded up to an integer to determine which substance is used in the objective function. 

In the present work, 19 kinds of pure working fluids are selected according to their critical temperature. We divided these working fluids into two groups to form the mixed working fluids randomly by the multi-objective algorithm. Table 2 shows the critical temperature and pressure of these working fluids.

**Figure 2:**
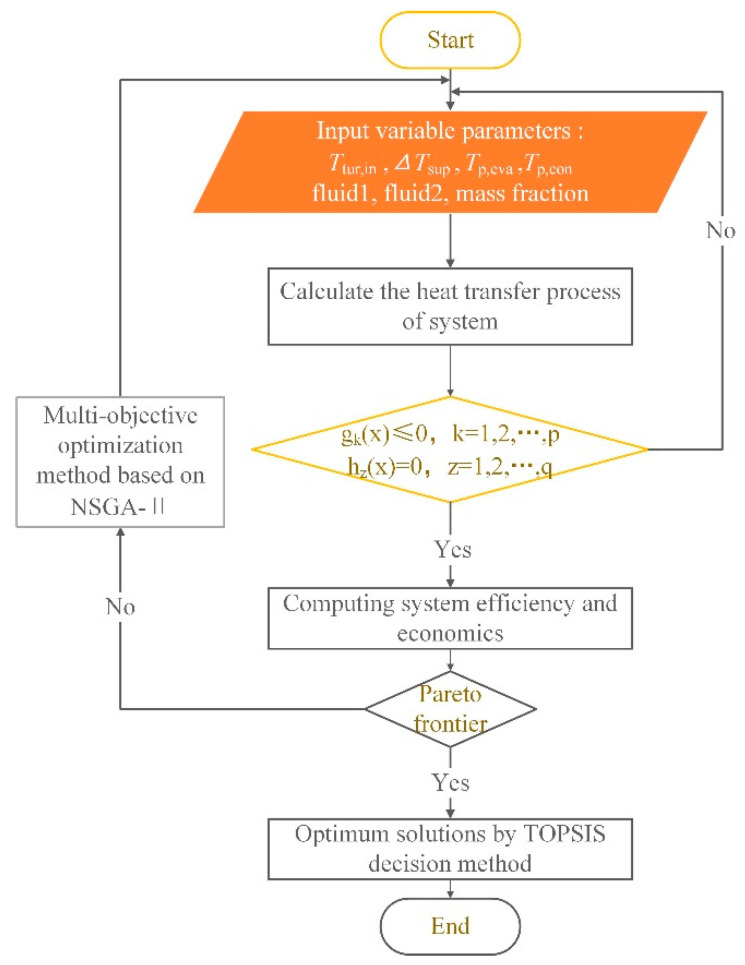
Flow chart of the optimization.

Accordingly, seven system parameters are selected as decision variables in the present work, including the inlet temperature of the turbine, superheat degree, PPTD in evaporator and condenser, the composition of the zeotropic mixture (fluid-1 and fluid-2) and mass fraction of the zeotropic mixture. The population size is 100, and the maximum generation is 70 in the NSGA-II method. The range of decision variables are shown in Table 3. The flow chart of the optimization is given in Figure 2. 

## 4. Results and Discussion

### 4.1. Pareto Frontier Solutions of the BORC System

#### 4.1.1. Pareto Frontier Solutions and Effect of Objectives on the Working Fluid Selection

In this work, two sets of optimization objectives, exergy efficiency vs. thermal efficiency, and exergy efficiency vs. LEC, are utilized to compare the effect of the objectives on the optimization of design parameters and the selection of working fluids. The thermal efficiency, exergy efficiency, and LEC represent the first law and second law thermodynamic indicators, and the thermo-economic indicator. Three heat source temperatures are considered: 423.15 K to 523.15 K with the step of 50 K. The condensation temperature is kept at 308.15 K. 

**Figure 3:**
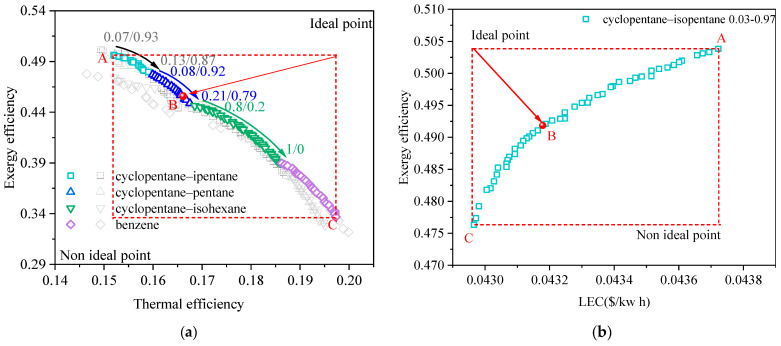
Pareto frontier solutions of the BORC system at a heat source temperature of 473.15 K for (**a**) exergy efficiency vs. thermal efficiency, and (**b**) exergy efficiency vs. LEC.

Figure 3 shows the Pareto frontier solutions of the BORC system at a heat source temperature of 473.15 K for (a) exergy efficiency vs. thermal efficiency and (b) exergy efficiency vs. LEC. Figure 3a exhibits how the Pareto frontier solutions of various working fluids are generated. For the normal multi-objective optimization of the ORC system, the working fluid is specified. Only the concentration of the mixture (fluid-1 and fluid-2) is the decision variable. In this case, we can obtain a set of Pareto frontier solutions for each specified working fluid, represented by the several types of scatters in light gray in Figure 3a. In this work, the selection of working fluid is integrated with the multi-objective optimization algorithm by adding two new decision variables. As a result, only one set of Pareto frontier solutions was achieved, shown as the colored scatter. It is seen that these solutions are contributed from the pieces of Pareto frontier solutions of each specified working fluid (light gray scatters). The combinations of working fluids and other decision variables which can provide relatively optimal solutions are screened out. These solutions are the final Pareto optimal solutions, and the other solutions are neglected during the optimization of the NSGA-II method integrated with working fluid selection. 

As listed in Table 2, ten types of pure working fluids are used for the fluid-1 group and nine for the fluid-2 group. Theoretically, we have ninety pairs of mixture candidates for the NSGA-II. The results in Figure 3a show that three pairs of mixtures and one pure working fluid were finally screened out as suitable working fluids. The concentration of mixtures for each solution was different. It varied to have fewer volatile components with the increase in thermal efficiency and the decrease in exergy efficiency. This indicates that the working fluid with higher critical temperature is beneficial to the system thermal efficiency but harmful to the exergy efficiency. The Pareto frontier exhibited a clear trade-off between exergy efficiency and thermal efficiency. The fluid properties and results at featured points of the Pareto frontier are given in Table 4 and Table 5, respectively. Point A used cyclopentane/isopentane as a working fluid and generated the highest exergy efficiency while providing the lowest thermal efficiency. From point A to point C, the exergy efficiency decreased from 49.6% to 33.7% while the thermal efficiency increased from 15.2% to 19.7%. Although the condensation temperature glide in Table 4 also varied, results in Figure 3a show that the thermodynamic performance of the BORC system is primarily affected by the critical temperature. According to Equations (8) and (9), the heat source exergy is fixed for a specified heat source temperature, and the exergy efficiency of the cycle is in proportion to the net power of the cycle. Consequently, point A in Figure 3a provides the maximal net output power of the system and the highest heat utilization rate of the heat source. On the contrary, point C has the lowest net output power and utilization rate of the heat source although it showed the highest thermal efficiency. Point B chosen by the TOPSIS method showed a balance between the exergy efficiency and thermal efficiency. At this point, the ORC system had an exergy efficiency of 45.6% and a thermal efficiency of 16.6% by using cyclopentane/pentane (0.17/0.83) as the working fluid. 

The mechanism of how to generate the Pareto frontier solution including the selection of working fluids has been explained in the analysis of Figure 3a. The Pareto frontier in other figures in the present work are all achieved through the same method. Figure 3b shows the Pareto frontier when the decision indicators are chosen as exergy efficiency and LEC. Compare the results in Figure 3a,b, we can see that the variation of the exergy efficiency and LEC in Figure 3b have a quite narrow range. From point A to point C, the exergy efficiency decreased from 50.4% to 47.6%, relatively 5.6% variation, and the LEC decreased from 0.0437 to 0.0429, relatively 1.8%. All of the solutions in Figure 3b are distributed around point A in Figure 3a. This phenomenon means that the two decision indicators, exergy efficiency and LEC, have quite a weak conflict with each other. This binary objective optimization tends to be the single-objective optimization. In our former work [18,19], we proposed the selection criteria for mixtures used for the ORC system based on the thermodynamic indicator of exergy efficiency. We found that when the ORC system had a high exergy efficiency, the LEC was relatively low. Those results are inconsistent with the present phenomenon. In the meantime, the results in Figure 3 also mean the exergy efficiency vs. LEC is not suggested for the multi-objective optimization as a strong indicator is preferred such as the exergy efficiency vs. thermal efficiency.

**Figure 4:**
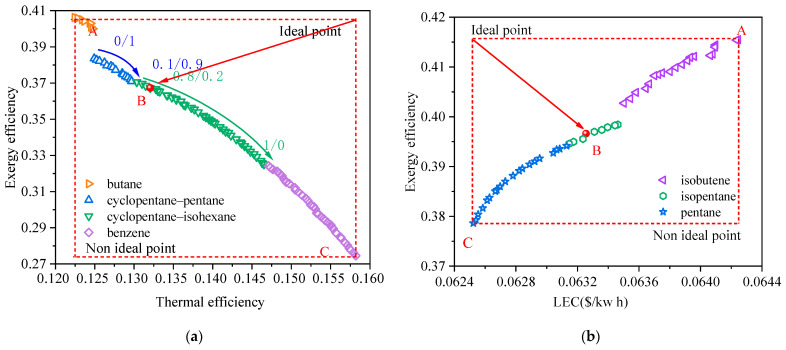
Pareto frontier solutions of the BORC system at a heat source temperature of 423.15 K for (**a**) exergy efficiency vs. thermal efficiency, and (**b**) exergy efficiency vs. LEC.

**Figure 5:**
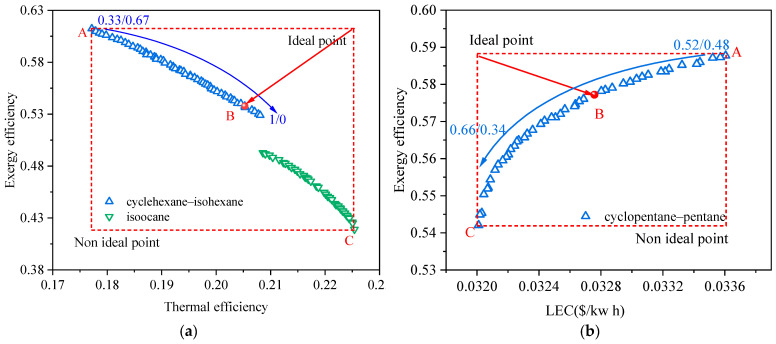
Pareto frontier solutions of the BORC system at a heat source temperature of 523.15 K for (**a**) exergy efficiency vs. thermal efficiency, and (**b**) exergy efficiency vs. LEC.

Figure 4 and Figure 5 show the Pareto frontier solutions of the BORC system at a heat source temperature of 423.15 K and 523.15K, respectively. The selected working fluid and results at featured points of the Pareto frontier are given in Table 6 and Table 7. It can be seen that the results in Figure 4 and Figure 5 showed similar trends to those in Figure 3. Different types of working fluid appeared at the Pareto frontier with varied concentrations. The working fluids with lower critical temperatures have higher exergy efficiency but lower thermal efficiency. In these three figures, the final determined point B of the solution for exergy efficiency vs. LEC approaches point A for exergy efficiency vs. thermal efficiency, reflecting the weak conflict between the exergy efficiency and LEC. The gaps at the Pareto frontier in Figure 4 and Figure 5a are caused by the significant change in the thermophysical properties with the change in working fluid types. As the heat source temperature becomes higher, the system exergy efficiency and the thermal efficiency also becomes higher while the LEC is decreased. The critical temperature of the selected working fluid for point B also goes higher. 

#### 4.1.2. Parametric Analysis of the Decision Variables

**Figure 6:**
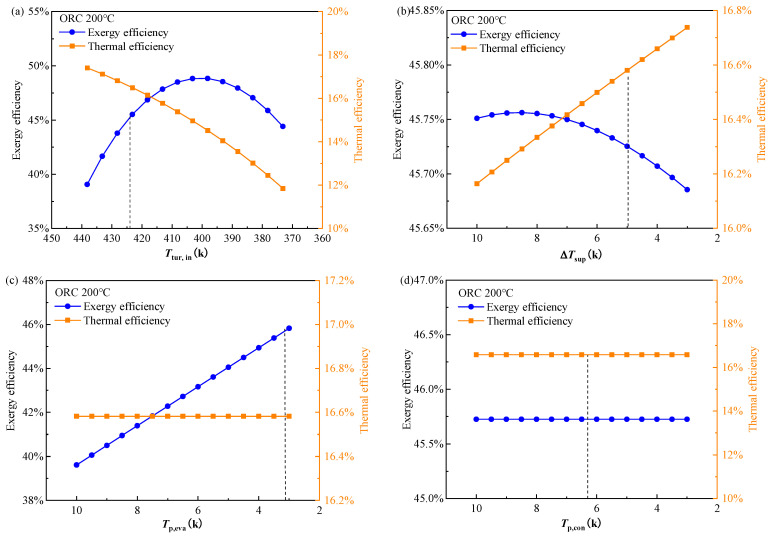

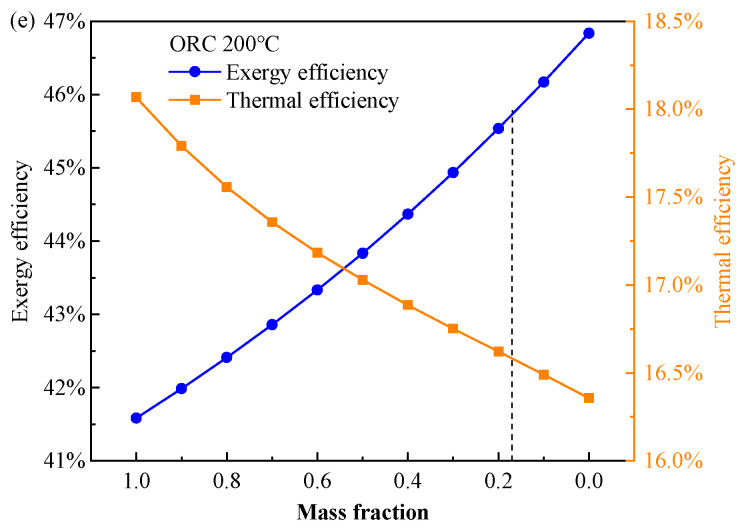
Effect of decision variables (**a**) turbine inlet temperature, (**b**) vapor superheat, (**c**) evaporator PPTD, (**d**) condenser PPTD, and (**e**) mixture mass fraction on exergy efficiency and thermal efficiency of the BORC system at a heat source temperature of 473.15 K.

**Figure 7:**
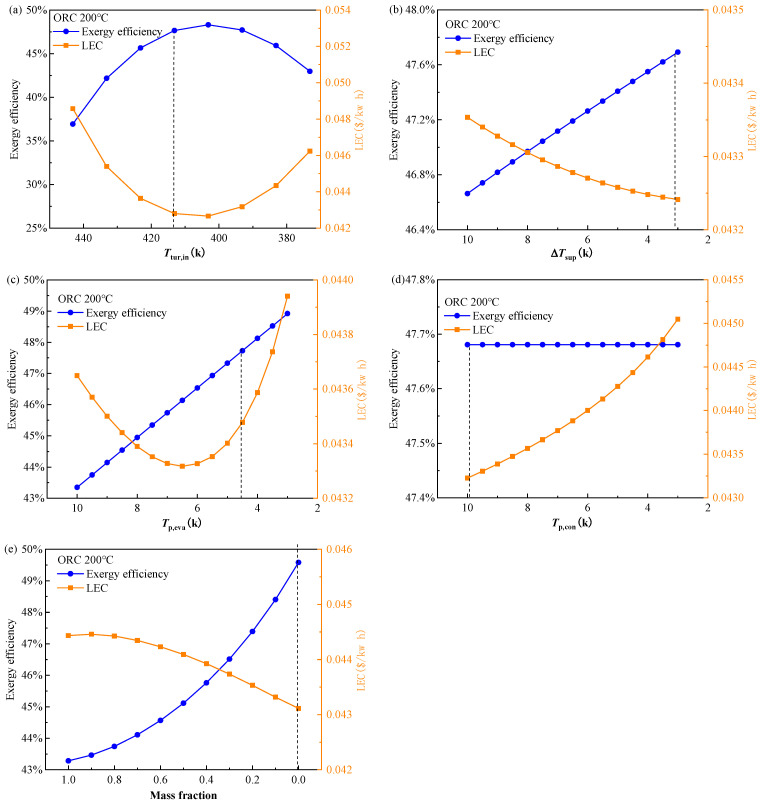
Effect of decision variables (**a**) turbine inlet temperature, (**b**) vapor superheat, (**c**) evaporator PPTD, (**d**) condenser PPTD, and (**e**) mixture mass fraction on exergy efficiency and LEC of the BORC system at a heat source temperature of 473.15 K.

In this section, the effect of the decision variables on the objectives is analyzed. The initial values of these variables are set as the values of point B in Table 5. When the effect of a specified decision variable is calculated, the other variables are kept unchanged. The calculation and analysis were carried out under the heat source temperature of 473.15 K. The results of exergy efficiency vs. thermal efficiency are given in Figure 6 and those of exergy efficiency vs. LEC are given in Figure 7. The dashed lines show the final determined values of point B in Table 5. The working fluid for results in Figure 6 is cyclopentane/pentane, thus, there are 5 decision variables left, that is the turbine inlet temperature $T_{\mathrm{tur},\mathrm{in}}$, the superheat of vapor $\Delta T_{\sup}$, PPTD of evaporator and condenser $T_{p,\mathrm{eva}}$, $T_{p,\mathrm{con}}$, and the mass fraction of the mixture working fluid. For the ORC system, both the exergy efficiency and thermal efficiency are expected to be high. It is seen in Figure 6 that these two objectives generally have a contrasting trend with the variation in the decision variables. The turbine inlet temperature is the most sensitive factor that influences the exergy and thermal efficiency. With the turbine inlet temperature decreased from 437.15 K to 373.15 K, the thermal efficiency decreases monotonically from 18% to 12% while the exergy efficiency first increased from about 38.5% to the maximum of 49% at 403.15 K, then decreased to 44.5%. As the superheat of vapor remains the same, the higher turbine inlet temperature represents the higher evaporation temperature which means the large enthalpy difference through the turbine. Thus, the higher turbine inlet temperature leads to higher thermal efficiency. However, the exergy efficiency is the combined result of the specific enthalpy difference through the turbine and the mass flow rate of the working fluid. A higher turbine inlet temperature results in a lower mass flow rate. Consequently, the exergy efficiency exhibits the parabola profile. 

The other four decision variables except the turbine inlet temperature have a relatively limited effect on the exergy and thermal efficiencies. In Figure 6b, the thermal efficiency increased with the reduction in vapor superheat while the exergy efficiency decreased. As the turbine inlet temperature is kept unchanged in this case, the higher vapor superheat degrees result in the lower evaporation temperature. Thus, the lower enthalpy difference through the turbine leads to lower thermal efficiency. In Figure 6c, as the turbine inlet temperature and vapor superheat are both fixed, the change in evaporator PPTD does not affect the cycle operation parameters. Hence, the thermal efficiency also remains unchanged. However, the decrease in the PPTD can increase the heat flux of the evaporator. As a result, the output work goes higher, and the exergy efficiency becomes higher. According to a similar mechanism, the change in the condenser PPTD does not affect the exergy efficiency and thermal efficiency, shown in Figure 6d, because the condensation temperature is set as a constant of 308.15 K during the calculation. In Figure 6e, with the decrease in mass fraction of cyclohexane in the mixture, the critical temperature of the working fluid becomes lower, leading to the reduction in the thermal efficiency and the increase in the exergy efficiency.

When the decision indicators are chosen as the exergy efficiency and LEC, the higher exergy efficiency and lower LEC are expected. Generally, it can be seen in Figure 7 that the effect of decision variables on these two objectives shows a consistent trend except for the evaporator PPTD. Combine this fact with the trend that the turbine inlet temperature is the most sensitive factor compared to the other four decision variables, the two indicators: exergy efficiency and LEC show quite weak conflict with each other, as mentioned in the Section 4.1.1. The results in Figure 7 can be strong evidence for the results in Figure 3b, Figure 4b and Figure 5b. The effect of the turbine inlet temperature on the exergy efficiency has been discussed above. The LEC is a comprehensive indicator, considering the cost and the output power of the ORC system. With the increase in exergy efficiency, both the cost and the output power increase, as a result, the LEC decreases. Figure 7a shows the well-coordinated trends between the exergy efficiency and LEC. The final determined turbine inlet temperature is located near the optimal point in the lines. The lower vapor superheat and higher condenser PPTD lead to the higher heat transfer temperature difference in the evaporator and condenser, respectively. Hence, the heat transfer area and the cost of heat exchangers are reduced which reduces the LEC. Thus, as is seen in Figure 7b,d, the final determining vapor superheat tends to be at the lower boundary of 3 K and the condenser PPTD approaches the upper boundary of 10 K. Although the effect of the evaporator PPTD on the LEC shows a ‘U’ type profile, the total variation range is quite narrow, relatively smaller than 1.6%, shown in Figure 7c. In Figure 7e, the reduction in the mass fraction of cyclopentane leads to the reduction in the mixture’s critical temperature. The LEC becomes higher, which means the output power of the ORC system increases. The cost of the system will also go higher, however, the LEC is reduced. As a result, the determined working fluid is a mixture of cyclopentane/isopentane with a mass fraction of 0.03/0.97, approaching the pure pentane. 

### 4.2. Pareto Frontier Solutions of the RORC System

#### 4.2.1. Pareto Frontier Solutions

**Figure 8:**
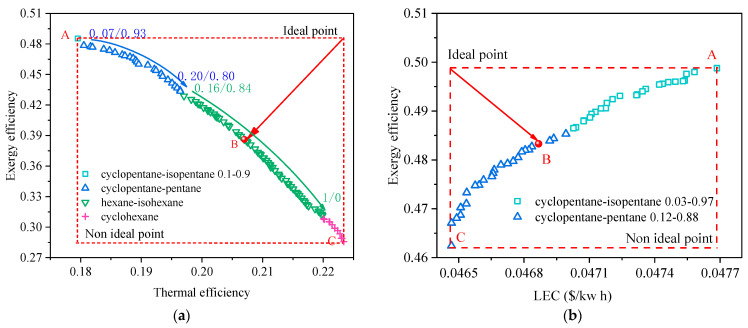
Pareto frontier solutions of the RORC system at a heat source temperature of 473.15 K for (**a**) exergy efficiency vs. thermal efficiency, and (**b**) exergy efficiency vs. LEC.

In this section, the multi-objective optimization of the RORC system and the selection of working fluids are discussed. The effectiveness of the regenerator is set as 0.8. Figure 8 shows the Pareto frontier solutions of the RORC system at a heat source temperature of 473.15 K for (a) exergy efficiency vs. thermal efficiency and (b) exergy efficiency vs. LEC. From a thermodynamic point of view, the RORC can generate the same amount of output work as the BORC while offering a relatively higher thermal efficiency if the same working fluid is adopted for both cycles. This is because the evaporation temperature/pressure and the condensation temperature/pressure remain the same in the RORC considering the only difference is that part of the heat absorbed from the heat source is now replaced by the same amount of heat released from the exhausted vapor of the turbine. In the present work, the exergy efficiency of the cycle is in proportion to the net power of the cycle for a specified heat source temperature. Thus, comparing the solutions on the Pareto frontier in Figure 3a and Figure 8a, we can find that the thermal efficiency corresponding to the same exergy efficiency becomes higher for the RORC. At point B in Figure 3a, the ORC system has an exergy efficiency of 45.6% and thermal efficiency of 16.6% while in Figure 8a the same exergy efficiency corresponds to the thermal efficiency of 19.3%. 

Generally, the results in Figure 8 exhibit similar trends to those in Figure 3. Several types of working fluid were selected on the Pareto frontier, including mixtures and pure substances. With the increase in thermal efficiency, the mass fraction of the less volatile component in the mixture becomes higher, which leads to an increase in the critical temperature of the mixture. In Figure 8a, the exergy efficiency varies from 28% to 48%, and the thermal efficiency varies from 18% to 22.3%. Comparably, the variation of exergy efficiency and LEC in Figure 8b is limited to a quite narrow range from 46.6% to 50.4% and from 0.0465 $/kWh to 0.0467 $/kWh, respectively. These results are consistent with those in Figure 3 and implied that the indicator of exergy efficiency vs. LEC would also make the binary objective optimization of the RORC tend to be a single objective optimization. It would lead to a set of decision variables generating a high exergy efficiency of the ORC system but low thermal efficiency. The information of optimal point B in Figure 8a,b are listed in Table 8. It is seen that the picked point B in Figure 8b has the exergy efficiency of 0.484, very close to point A in Figure 8a. However, the thermal efficiency of point A is 18%, lower than 21.1% at point B. 

Typically, the utilization of the IHE in the RORC will reduce the total heat transfer area of heat exchangers compared to that of the BORC due to the decreasing heat flux of the evaporator and condenser. However, it is seen in Figure 8b that the LEC of solutions on the Pareto frontier is higher than those in Figure 3b. The reason is that the bare module cost of buying one more heat exchange for the RORC is higher than the cost reduction contributed to the reduced heat transfer area. The final optimal point B of the RORC has the exergy efficiency of 48.4% and LEC of 0.0469 $/kWh, compared to the exergy efficiency of 49.2% and LEC of 0.0432 $/kWh for the BORC system.

#### 4.2.2. Parametric Analysis of the Decision Variables

**Figure 9:**
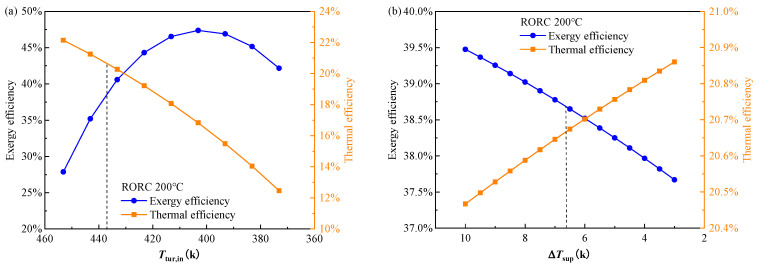

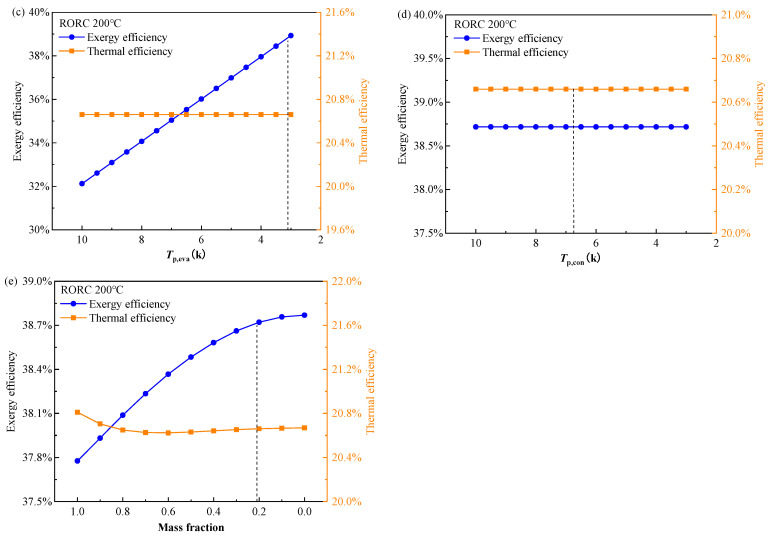
Effect of decision variables (**a**) turbine inlet temperature, (**b**) vapor superheat, (**c**) evaporator PPTD, (**d**) condenser PPTD, and (**e**) mixture mass fraction on exergy efficiency and thermal efficiency of the RORC system at a heat source temperature of 473.15 K.

**Figure 10:**
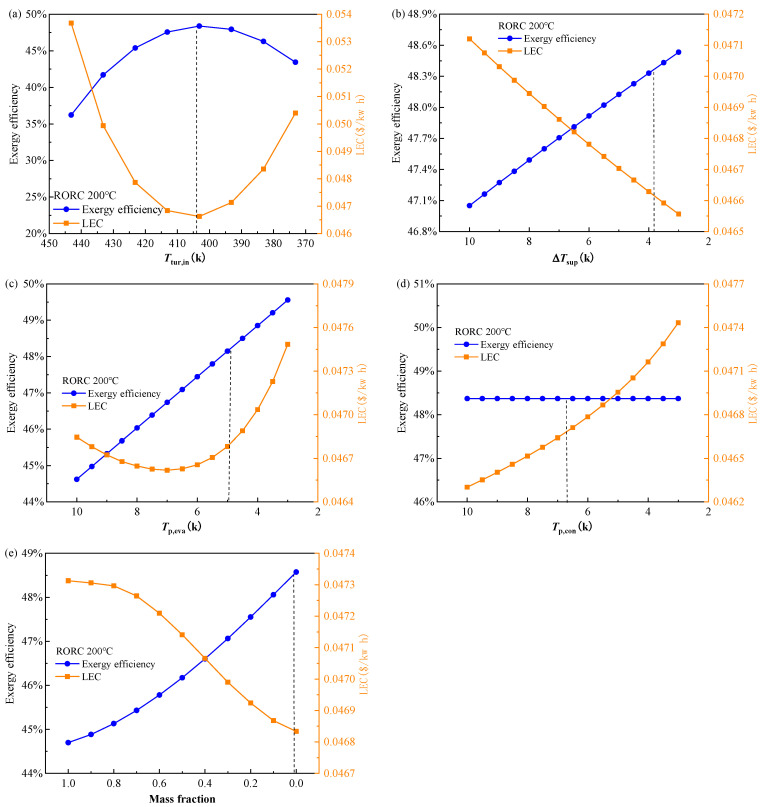
Effect of decision variables (**a**) turbine inlet temperature, (**b**) vapor superheat, (**c**) evaporator PPTD, (**d**) condenser PPTD, and (**e**) mixture mass fraction on exergy efficiency and LEC of the RORC system at a heat source temperature of 473.15 K.

Figure 9 and Figure 10 show the effect of the decision variables on the objectives of the RORC at the heat source temperature of 473.15 K. These variables are initialed as the values of point B in Table 8. In Figure 9, the decision indicators are exergy efficiency and thermal efficiency, which are both expected to be high for the RORC system. It can be seen that the results in Figure 9 have similar trends to those in Figure 6 and these two objectives generally have a strong conflict with each other. The higher vapor superheat degree, lower evaporator PPTD, and lower mass fraction of the less volatile component in the mixture will benefit the exergy efficiency but reduce or have no effect on the thermal efficiency. The turbine inlet temperature has a strong effect on the exergy and thermal efficiency while the other four decision variables exhibit relatively limited influence. With the turbine inlet temperature decreased from 453.15 K to 373.15 K, the exergy efficiency shows a significant increase from 27.5% to 47% at 403.15 K, then decreases to 42% while the thermal efficiency decreases monotonically from 22% to 12%. The mechanism of the results in Figure 9a is the same as that for the BORC, which has been explained in the discussion of Figure 6a. As the condensation temperature is set as a constant of 308.15 K during the calculation, the change in condenser PPTD does not affect the cycle operation parameters. Consequently, exergy efficiency and thermal efficiency in Figure 6d remain unchanged with the variation of condenser PPTD. 

Figure 10 shows the effect of decision variables on the exergy efficiency and LEC. As mentioned above, these two indicators have a quite weak conflict with each other, reflected in Figure 10; it is seen that exergy efficiency and LEC mainly show a consistent trend. The higher exergy efficiency corresponds to the lower LEC. The turbine inlet temperature is still the most sensitive factor compared to the other four decision variables. The perfect consistent variation of exergy efficiency and LEC can be seen in Figure 10a. With the turbine inlet temperature decreased from 453.15 K to 373.15 K, the exergy efficiency increased from 36% to 48% at 403.15 K, then decreased to 43% while the LEC decreases from 0.0535 $/kWh to 0.0465 $/kWh, then increase to 0.0505 $/kWh. The final determined turbine inlet temperature of point B is located at the optimal point in the lines, corresponding to the highest exergy efficiency and lowest LEC. The lower vapor superheat, lower evaporator PPTD and lower mass fraction of the less volatile component in the mixture impose a positive effect on the exergy efficiency but a negative effect on the LEC at the same time. As the condensation temperature is kept at 308.15 K during the calculation, the change in the condenser PPTD does not affect the cycle operation parameters. Thus, exergy efficiency in Figure 10d is unchanged with the variation in the condenser PPTD. However, the larger condenser PPTD could result in a smaller heat transfer area according to the heat transfer mechanism. Consequently, the LEC gradually decreases with the increase in the condenser PPTD. 

## 5. Conclusions

In the present study, a multi-objective optimization based on the NSGA-II and TOPSIS decision-making method is carried out for the BORC and RORC systems. An approach to integrate the screening of working fluid including both the pure substance and mixture with multi-objective optimization is proposed. Two sets of decision indicators, exergy efficiency vs. thermal efficiency and exergy efficiency vs. LEC, are adopted. Five decision variables including the turbine inlet temperature, vapor superheat degree, the evaporator and condenser pinch temperature difference, and the mass fraction of the mixture are considered in the NSGA-II. Their effect on the objectives is examined. The main conclusions are drawn as follows:

(1) The selection of working fluid and multi-objective optimization of the cycle parameters could be realized simultaneously by parameterizing pure working fluids into arrays of numbers. Several types of the working fluid, pure or mixed, are presented on the Pareto frontier;
(2) The turbine inlet temperature is the most effective factor for both the BORC and RORC systems while the other four decision variable has quite limited influence on the objectives. The nonlinear relation between the exergy efficiency and the turbine inlet temperature is observed;
(3) The decision variables mainly impose a reverse effect on the exergy efficiency and thermal efficiency while the exergy efficiency and LEC exhibit quite a weak conflict with each other. This makes the binary objective optimization tend to be a single objective optimization when the objectives are set as exergy efficiency and LEC;
(4) The RORC with an IHE can provide higher thermal efficiency than ORC at the same exergy efficiency while the LEC of the RORC system also becomes higher because the bare module cost of buying one more heat exchange for the RORC is higher than the cost reduction contributed to the reduced heat transfer area;
(5) The Pareto frontier solution is distributed in similar trends at different heat source temperatures. Under the heat source temperature of 423.15 K, the final obtained exergy efficiency and thermal efficiencies are 45.6% and 16.6% for BORC, and 38.6% and 20.7% for RORC, respectively.

## Figures and Tables

**Table 1:** The costing coefficient of the components.

Coefficient	Heat Exchanger	Pump	Expander
$K_{1}$	4.325	3.389	3.514
$K_{2}$	−0.303	0.054	0.598
$K_{3}$	0.163	0.154	0
$B_{1}$	1.63	1.89	
$B_{2}$	1.66	1.35	
$C_{1}$	0.039	−0.394	
$C_{2}$	−0.113	0.396	
$C_{3}$	0.082	−0.002	
$F_{m}$	1.35	1.55	
$F_{\mathrm{bm}}$			1.5

**Table 2:** Working fluid candidates for the multi-objective optimization of the BORC and RORC system.

Fluid 1 (Number)	Critical Temperature (K)	Critical Pressure (MPa)	Fluid 2 (Number)	Critical Temperature (K)	Critical Pressure (MPa)
hexane (1)	507	3.03	propyne (1)	402	5.63
acetone (2)	508	4.70	isobutane (2)	407	3.63
cyclopentane (3)	511	4.57	isobutene (3)	418	4.01
heptane (4)	540	2.74	butene (4)	419	4.00
isooctane (5)	544	2.57	butane (5)	425	3.79
cyclohexane (6)	553	4.08	neopentane (6)	433	3.19
benzene (7)	562	4.91	isopentane (7)	460	3.37
octane (8)	569	2.50	Pentane (8)	469	3.37
nonane (9)	594	2.28	isohexane (9)	497	3.04
toluene (10)	591	4.13			

**Table 3:** The boundaries of decision variables.

Item	Symbol	Unit	Range
Inlet temperature of the turbine	$T_{\mathrm{tur},\mathrm{in}}$	K	373–453
Superheat degree	$\Delta T_{\sup}$	K	3–10
PPTD in evaporator	$T_{p,\mathrm{eva}}$	K	3–10
PPTD in condenser	$T_{p,\mathrm{con}}$	K	3–10
Fluid-1	F1		1–10
Fluid-2	F2		1–9
Mass fraction	x		0–1

**Table 4:** Suitable working fluid at the Pareto frontier at a heat source temperature of 473.15 K.

	Selected Working Fluid	Concentration	Critical Temperature (K)	Condensation Temperature Glide (K)
Figure 3a:	cyclopentane/isopentane	0.07/0.93→0.13/0.87	464→467	0.8→1.45
	cyclopentane/pentane	0.08/0.92→0.21/0.79	473→479	0.25→0.69
	cyclopentane/isohexane	0.8/0.2→1/0	509→511	0.3→0
	benzene	1/0	562	0
Figure 3b:	cyclopentane/isopentane	0.03/0.97	462	0.35

**Table 5:** The results of featured points at the Pareto frontier at a heat source temperature of 473.15 K.

	Point	Exergy Efficiency	Thermal Efficiency	LEC ($/kWh)	$T_{\mathrm{tur},\mathrm{in}}$ (K)	$\Delta T_{\sup}$ (K)	$T_{p,\mathrm{eva}}$ (K)	$T_{p,\mathrm{con}}$ (K)	Working Fluid
Figure 3a:	A	0.496	0.152		411	4.86	3.12	5.56	cyclopentane/isopentane (0.07/0.93)
	B	0.456	0.166		424	4.97	3.12	6.30	cyclopentane/pentane (0.17/0.83)
	C	0.337	0.197		435	5.23	3.76	7.52	benzene
Figure 3b:	A	0.504		0.0437	414	3.08	3.09	9.75	cyclopentane/isopentane (0.03/0.97)
	B	0.492		0.0432	413	3.08	4.56	9.91	cyclopentane/isopentane (0.03/0.97)
	C	0.476		0.0429	413	3.11	6.41	9.88	cyclopentane/isopentane (0.03/0.97)

**Table 6:** Suitable working fluid at the Pareto frontier at a heat source temperature of 423.15 K.

	Point	Exergy Efficiency	Thermal Efficiency	LEC ($/kWh)	$T_{\mathrm{tur},\mathrm{in}}$ (K)	$\Delta T_{\sup}$ (K)	$T_{p,\mathrm{eva}}$ (K)	$T_{p,\mathrm{con}}$ (K)	Working Fluid
Figure 4a:	A	0.406	0.122		381	4.21	3.17	6.58	butane
	B	0.368	0.131		381	4.60	3.04	7.30	cyclopentane/isohexane (0.83/0.17)
	C	0.274	0.158		397	3.95	3.13	7.93	benzene
Figure 4b:	A	0.415		0.0642	372	3.33	3.29	9.68	isobutene
	B	0.399		0.0632	372	3.33	3.43	9.83	isopentane
	C	0.378		0.0625	371	3.35	5.34	9.88	pentane

**Table 7:** Suitable working fluid at the Pareto frontier at a heat source temperature of 523.15 K.

	Point	Exergy Efficiency	Thermal Efficiency	LEC ($/kWh)	$T_{\mathrm{tur},\mathrm{in}}$ (K)	$\Delta T_{\sup}$ (K)	$T_{p,\mathrm{eva}}$ (K)	$T_{p,\mathrm{con}}$ (K)	Working Fluid
Figure 5a:	A	0.607	0.177		435	3.14	3.02	5.81	cyclopentane/pentane (0.33/0.67)
	B	0.529	0.207		458	3.44	3.04	5.72	cyclopentane/pentane (0.98/0.19)
	C	0.414	0.225		470	3.68	3.77	5.71	benzene
Figure 5b:	A	0.588		0.0336	442	3.42	3.16	8.11	cyclopentane/pentane (0.52/0.48)
	B	0.577		0.0328	442	3.37	4.63	8.47	cyclopentane/pentane (0.52/0.48)
	C	0.536		0.0320	443	3.44	7.28	8.92	cyclopentane/pentane (0.66/0.34)

**Table 8:** Suitable working fluid at the Pareto frontier of the RORC system at a heat source temperature of 473.15 K.

	Point	Exergy Efficiency	Thermal Efficiency	LEC ($/kWh)	$T_{\mathrm{tur},\mathrm{in}}$ (K)	$\Delta T_{\sup}$ (K)	$T_{p,\mathrm{eva}}$ (K)	$T_{p,\mathrm{con}}$ (K)	Working Fluid
Figure 8a:	A	0.485	0.180		415	4.97	3.60	7.37	cyclopentane/isopentane (0.1/0.9)
	B	0.386	0.207		437	6.76	3.22	6.87	hexane/isohexane (0.21/0.71)
	C	0.286	0.223		447	7.62	3.62	5.30	cyclohexane
Figure 8b:	A	0.499		0.0477	406	3.90	4.07	5.46	cyclopentane/isopentane (0.03/0.97)
	B	0.484		0.0469	404	3.82	4.69	5.63	cyclopentane/pentane (0.04/0.96)
	C	0.462		0.0465	403	3.95	7.15	5.73	cyclopentane/pentane (0.12/0.88)

## Nomenclature

A	$\mathrm{heat}\;\mathrm{transfer}\;\mathrm{area}\;(m^{2}$)
Bo	boiling number
C	cost ($)
CRF	capital recovery cost
d	diameter of the heat exchanger tube (m)
$\dot{E}$	exergy (kw)
$F_{\mathrm{bm}}$	bare module factor
$F_{m}$	material factor
$F_{p}$	pressure factor
*h*	specific enthalpy (kJ/kg)
K	$\mathrm{overall}\;\mathrm{heat}\;\mathrm{transfer}\;\mathrm{coefficient}\;(W/m^{2}$ K)
LEC	levelized energy cost ($/kWh)
$\dot{m}$	mass flow rate (kg/s)
Nu	Nusselt number
P	pressure (kPa)
Pr	Prandtl number
Q	energy (kW)
Re	Reynolds number
s	specific entropy (kJ/kg K)
T	temperature (K)
$t_{op}$	operation hour (h)
W	power (kW)
**Greek Symbols**	
α	$\mathrm{forced}\;\mathrm{convection}\;\mathrm{heat}\;\mathrm{transfer}\;\mathrm{coefficient}\;(W/m^{2}$ K)
λ	Heat conductivity (W/m K)
η	efficiency
$\Delta T_{\mathrm{con}}$	condensation temperature glide (K)
$\Delta T_{\mathrm{evp}}$	boiling process temperature glide (K)
ρ	$\mathrm{density}\;(\mathrm{kg}/m^{3}$)
**Subscript**	
0	environment, in equilibrium with the environment
con	condensation, condenser
eva	evaporator
h	heatsource
in	inlet
net	Net
o	outlet
p	pinch point temperature difference
pump	pump
reg	regenerator
s	isentropic
sup	degree of superheat
tur	turbine
v	vapor
wf	working fluid
